# Survivin Delta Ex3 Overexpression in Thyroid Malignancies

**DOI:** 10.1371/journal.pone.0100534

**Published:** 2014-06-19

**Authors:** Joanna Waligórska-Stachura, Mirosław Andrusiewicz, Nadia Sawicka-Gutaj, Maciej Biczysko, Anna Jankowska, Marta Kubiczak, Agata Czarnywojtek, Elżbieta Wrotkowska, Marek Ruchała

**Affiliations:** 1 Department of Endocrinology, Metabolism and Internal Medicine, Poznan University of Medical Sciences, Poznań, Poland; 2 Department of Cell Biology, Poznan University of Medical Sciences, Poznań, Poland; 3 Department of General, Gastroenterological and Endocrine Surgery, Poznan University of Medical Sciences, Poznań, Poland; The Chinese University of Hong Kong, Hong Kong

## Abstract

**Context:**

Thyroid cancer incidence has increased significantly during the past decades and is the most common type of endocrine malignancy. Many factors in thyroid cancers were studied as independent predictors of a poor prognosis.

**Objective:**

The objective of the study was to evaluate survivin expression – *BIRC5* and its splice variants: survivin delta Ex3 and survivin 2B in benign and malignant thyroid nodules.

**Design:**

Thyroid tissues samples from a group of 50 patients consisting of: 29 patients with thyroid cancers (including medullary, papillary, follicular and undifferentiated types), as well as from 21 patients with non-cancerous thyroid tissues (including: 11 benign thyroid lesions and 10 healthy thyroid samples).

**Main Outcome Measures:**

The analysis of the survivin gene expression and evaluation of the level of splice variants were performed using quantitative RT-PCR.

**Results:**

A statistically significant higher level of expression of survivin gene – *BIRC5* was detected in thyroid malignant nodules, when compared with benign lesions and healthy thyroid samples. Moreover, the comparison of survivin relative expression in different staged tumors (pT1, pT3, and pT4) revealed a much higher amount of *BIRC5* transcripts in tumor tissues of pT3/pT4. The comparison of survivin expression between benign thyroid nodules and healthy thyroid did not reveal significant differences. Importantly, high expression rate of the survivin delta Ex3 splice variant characterized thyroid carcinomas.

**Conclusion:**

The results suggest that survivin, especially survivin delta Ex3 splice variant being overexpress, is a characteristic feature of thyroid malignancy.

## Introduction

Thyroid cancer is the most common type of endocrine malignancy [1]. Well-differentiated thyroid cancers arise from follicular cells and encompass papillary, follicular and Hürthle cell carcinomas. Several different tumor-associated antigens have been described for endocrine malignancies. Clinical and histopathological parameters affecting the course of disease and patient prognosis such as male gender, advanced age, large tumor size, differentiation status and lymph node or distant metastases, have been studied for thyroid cancer [2]. The best known molecules involved in thyroid tumorigenesis include those of RTK/RAS/BRAF/MAP kinase pathway, which is suggested to be involved in the development of papillary thyroid carcinoma (PTC) - the most common type of thyroid cancer. In fact, RAS oncogenes were the first ones to be associated with thyroid cancer, and their mutations were found in benign and malignant follicular neoplasms and in follicular variant PTC [1]. The BRAF mutations are the most common genetic disorder in PTC, found in about 83% of studied cases [3]. However, there are mutations in other genes such as: proto-oncogene RET, MET and VEGFR leading to medullary thyroid cancer [4].

Recently, survivin gene polymorphism and its expression as a prognostic value in thyroid cancer was postulated [5], [6], [7]. Survivin is an anti-apoptotic protein abundantly expressed in a variety of neuroendocrine cancer cells, including pheochromocytoma and GEP tumor cells [8], [9]. Its presence was shown also in various human benign neoplasms, such as breast adenomas, Bowen’s disease, colon polyps and benign tumors, derived from the nervous system [10], [11], [12], [13]. In malignant tumors, survivin expression correlates with poor prognosis, local recurrence and shorter, disease-free survival [14], [15], [16], [17], [18], [19]. Therefore, it is suggested that survivin may be a negative prognostic marker in the majority of human cancers.

Survivin is the smallest mammalian member of the inhibitor-of-apoptosis gene family with bifunctional roles [20], [21], [22], [23]. It is expressed in proliferating cells, exclusively in the G2 and M phases, but its over-expression in cancer cells is observed throughout the whole cycle [22], [23]. Apart from full-length survivin, which is derived from exons 1–4, four splice variants of the gene have additionally been characterized: survivin 2B, survivin 3B, survivin 2 Alpha and survivin delta Ex3 [24], [25]. Many reports suggest that survivin delta Ex3 and survivin 3B are cytoprotective proteins, while survivin 2 Alpha and survivin 2B have pro-apoptotic properties. Survivin delta Ex3 has also been associated with higher tumor staging, increased tumor aggressiveness, as well as poor prognosis in different human malignancies [26]. Therefore, for many malignant tumors, survivin has been suggested as a new target for anti-cancer therapy. The early results of studies using survivin antagonists [the antisense oligonucleotides, ribozymes, small interfering RNAs, or immunotherapy (survivin-based vaccines)], are promising [27].

Thus, we decided to determine survivin and its splice variants: survivin 2B, survivin delta Ex3 expression in various malignant and benign thyroid lesions, by reverse transcription followed by real-time quantitative PCR (RT-qPCR).

## Materials and Methods

With approval from the local institutional review board and the ethics committee of Poznan University of Medical Sciences, tissue samples were obtained from 50 patients who had undergone thyroid removal. All participants provided informed written consent. Tissues of all the patients were obtained following surgery performed between 2012 and 2013 at the Department of General, Gastroenterological and Endocrine Surgery, Poznan University of Medical Sciences in Poznan, Poland.

Resected thyroid tissues were immediately stored in RNAs protective medium – RNA*Later* (Sigma Aldrich). The studied group consisted of 29 tissue samples of thyroid cancers including: 19 cases of PTC, 4 cases of undifferentiated thyroid cancer, 4 cases of medullary thyroid cancer, and 2 cases of follicular thyroid cancer (Table 1). According to the TNM classification for thyroid cancer, the examined group included 12 cases of pT3 or 4 and 17 cases of pT1 or pT2. In pT1 staged cancers we found three microcarcinomas. The second group of examined tissues consisted of 21 samples of non-cancerous thyroid tissues. This group was further subdivided into benign thyroid nodules: 4 cases of follicular adenomas, 3 cases of hyperplastic thyroid nodules, 4 cases of colloid nodules and 10 cases of healthy thyroid tissues (from healthy regions of glands removed for thyroid cancer or goiter).

**Table 1:** Clinical characteristics of patients with thyroid carcinoma.

No. of patients	Age	Gender	Tumor Status/size	Nodal status	Metastatic status	Pathologic subtype	Multifocalty
1	61	M	pT4	N0	M0	UTC	Yes
2	81	F	pT3	N0	M0	PTC	Yes
3	29	F	pT4a	N1	M0	PTU	Yes
4	31	F	pT4a	N1	M0	PTU	Yes
5	28	F	pT2	N1	M0	PTU	Yes
6	62	M	pT3	N0	M0	PTU	No
7	64	M	pT2	N0	M0	PTU	No
8	50	F	pT4	N1	M1	UTC	Yes
9	56	F	pT1a	N0	M0	PTU	No
10	59	F	pT1b	N0	M0	PTU	No
11	79	F	pT1b	N0	M0	PTU	No
12	68	F	pT4	N0	M0	UTC	No
13	38	F	pT1b	N0	M0	PTU	No
14	61	M	pT4	N1	M0	MTC	Yes
15	78	F	pT4	N1	M1	UTC	Yes
16	63	M	pT1b	N0	M0	FTC	No
17	46	M	pT3	N1	M0	MTC	Yes
18	63	M	pT1b	N0	M0	FTC	No
19	79	F	pT1	N0	M0	PTC	No
20	74	F	pT1b	N0	M0	MTC	Yes
21	68	M	pT4	N1	M0	MTC	Yes
22	57	F	pT1b	N0	M0	PTC	No
23	32	M	pT2	N0	M0	PTC	No
24	63	M	pT1b	N0	M0	PTC	No
25	67	M	pT4	N1	M0	PTU	Yes
26	37	F	pT1a	N0	M0	PTU	No
27	42	F	pT1a	N0	M0	PTU	No
28	42	F	pT2	N1	M0	PTU	Yes
29	28	F	pT1a	N0	M0	PTU	No

PTC, papillary thyroid cancer, FTC, follicular thyroid cancer, UTC undifferentiated thyroid cancer, MTC, medullary thyroid cancer.

In the examined thyroid cancers group, we found 10 cases with the presence of lymph node metastases, two cases with distal metastases and 13 cases manifesting multifocality.

The patients’ mean age at the moment of thyroid surgery was 54.5 years (aged 28–81 yrs). 19 patients were male (aged 27–65 yrs) and 31 were female (aged 28–81 yrs).

### RNA Isolation

Total cellular RNA was extracted using the TriPure Isolation Reagent (Roche Diagnostic), according to the manufacturer’s protocol. The concentration of total RNA was determined spectrophotometrically and electrophoretically.

### Reverse Transcription and Quantitative PCR (RT-qPCR)

In order to synthestise cDNA one microgram of total RNA was used as a template for reverse transcription using the oligo(dT)_10_ primer and Transcriptor Reverse Transcriptase (Roche Diagnostics) according to the manufacturer’s protocol. As a negative control, a “no template control” (NTC) was used, in which reverse transcriptase was replaced with water in the reaction mixture. qPCR assays were designed to enable amplification of: BIRC5 [NCBI: NM_001168], BIRC5-ΔEx3 [NCBI: NM_001012270.1], BIRC5-2B [NCBI: NM_001012271.1] as well as housekeeping gene – HPRT [Human HPRT Gene Assay Cat. No. 05 046 157 001 (Roche Diagnostics)] The nucleotide sequences of primers and hybridization probes are shown in Table 2. qPCR was performed using the TaqMan Master Reagents Kit (Roche Diagnostics). Experiments were performed according to the delivered protocol. PCR efficiencies were calculated from the standard curves, which were generated using serial dilutions of cDNA library constructed from OVCAR3 cell line. Relative expression of analyzed genes was normalized against the phosphoribosylotransferase (HPRT) housekeeping gene (Human HPRT Gene Assay, Roche Diagnostics). Each reaction sets involved template control (NTC, negative control.

**Table 2:** Primers and hydrolysis probes used in real-time PCR.

Gene	TaqMan probe No	forward primer 5′→3′	reverse primer 5′→3′	amplicon length
total BIRC5	#36 (Cat. No. 04687949001)	gcccagtgtttcttctgctt	aaccggacgaatgcttttta	88 bp
BIRC5-ΔEx3	#36 (Cat. No. 04687949001)	cagtgtttcttctgcttcaagg	cttattgttggtttcctttgcat	77 bp
BIRC5-2B	#36 (Cat. No. 04687949001)	tctgcttcaaggagctgga	aaagtgctggtattacaggcgta	88 bp
HPRT	Human HPRT Gene Assay, Cat. No. 05 046 157 001 (Roche Diagnostics)			

### Data Collection and Statistical Analysis

All experiments were performed in triplicates, using independently synthesized cDNA. qPCR data was assembled using the LightCycler software 4.05 dedicated for the LightCycler 2.0 (Roche Diagnostics). The obtained data was used in statistical analyses.

Tests were considered to be statistically significant if the *P*-value was lower than 0.05. Statistical analyses were performed with MedCalc version 12.1.3.0 (MedCalc Software). A comparison of the analyzed parameters between two groups was performed with the Mann-Whitney test.

## Results

To evaluate the expression levels of survivin and its splice variants (survivin 2B and survivin delta Ex3) in malignant thyroid nodules and non-cancerous thyroid tissues, total RNAs from thyroid tissues were isolated and subjected to RT-qPCR.

**Figure 1:**
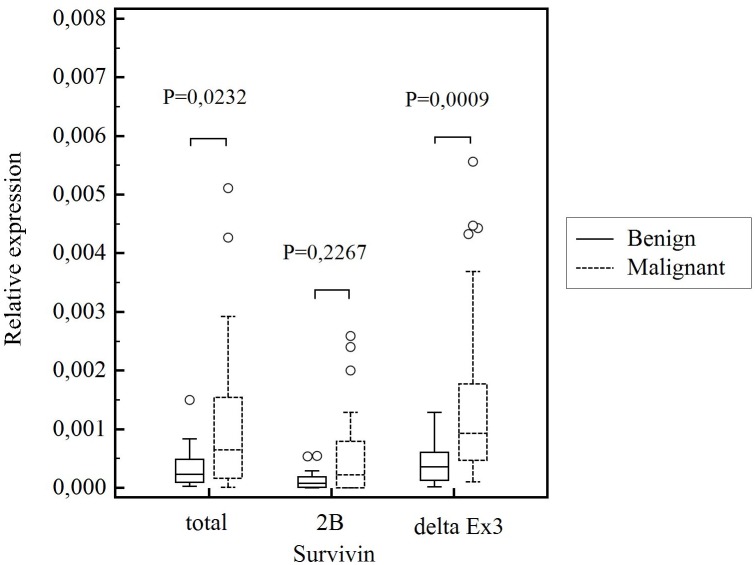
Comparison of relative survivin gene expression, survivin 2B expression and survivin delta Ex3 expression in benign and malignant thyroid nodules. Central box represents the values from the lower to upper quartile (25^th^ to 75^th^ percentile). The middle line represents the median. The thin vertical lines extending up or down from the boxes to horizontal lines (so-called whiskers) extend to a multiple of 1,5× the distance of the upper and lower quartile, respectively. Outliers are any values beyond the whiskers.

The results of the study (presented in Fig. 1) showed that survivin gene *BIRC5* was expressed at significantly higher levels in the thyroid cancers (*P* = 0,0232), than in benign thyroid nodules. Similar results were obtained when survivin delta Ex3 splice variant expression was evaluated. The amount of this splice variant’s transcript was higher in malignant thyroid nodules than in benign thyroid tissues and the difference was observed to be statistically significant (*P* = 0,0009) (Fig. 1). There was no statistically significant difference (*P* = 0,2267) in survivin 2B expression between benign and malignant thyroid nodules (Fig. 1).

Survivin and its splice variants mRNA level was also detected at higher levels in thyroid cancers when compared with healthy thyroid tissues (for survivin, *P* = 0,0127; survivin 2B, *P* = 0,0221; survivin delta Ex3, *P* = 0,0008). There was no significant difference between the amount of survivin and its splice variant transcripts in benign thyroid nodules and healthy thyroid samples (for survivin, *P* = 0,6313; survivin 2B, *P* = 0,1868; survivin delta Ex3, *P* = 0,6490).

**Figure 2:**
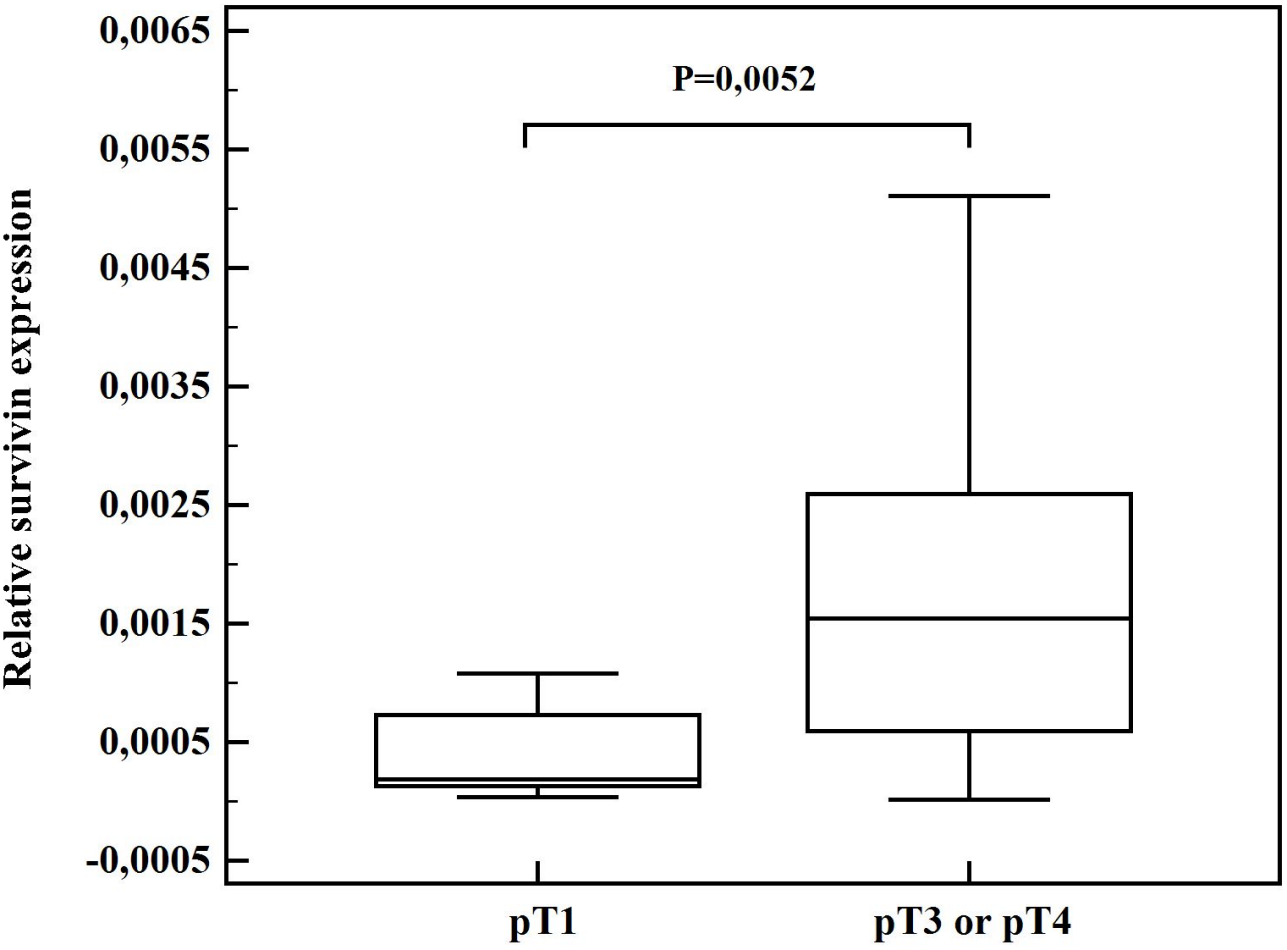
Comparison of relative survivin expression in tumors staged pT1 and pT3 or pT4. Central box represents the values from the lower to upper quartile (25^th^ to 75^th^ percentile). The middle line represents the median. The horizontal line extends from the minimum to the maximum value.

A comparison of survivin expression in different thyroid cancer samples classified according to the TNM staging system, revealed that survivin and its splice variant survivin 2B mRNA level (*P* = 0.0052) in tumors staged pT3 and pT4, are much higher than in pT1 tumors. A trend for overexpression of survivin delta Ex3 in tumors staged pT3/pT4 compared with pT1 was observed (*P = *0,0548) (Fig. 2).

There was no increase in survivin expression of multifocal thyroid cancers (for survivin, *P* = 0,1418; survivin 2B, *P* = 0,4360; survivin delta Ex3, *P* = 0,1592).

A significantly higher level of survivin delta Ex3 was observed in thyroid cancers manifestation of lymph node metastases (*P* = 0,0388). However, the level of survivin (*P* = 0,1305), and survivin 2B splice variant (*P* = 0,7247) showed no differences.

There was also no significant correlation found in relation to age and gender.

## Discussion

Despite the large body of research and well-defined clinical effective markers, many studies focus on the molecular features characterizing thyroid cancers. Recently, survivin overexpression was proposed to be a negative prognostic marker for variety of human cancers [28], [29]. Its presence was also documented in thyroid cancers. What it is more, survivin was shown to correlate with tumor aggressiveness and outcome [5], [24], [30].

Survivin has been postulated to be an unfavorable molecule for papillary thyroid cancer, because of the close correlation between high survivin expression and the presence of lymph node metastasis. It is suggested that evaluation of survivin expression in fine needle aspiration samples might be a useful tool for the identification of those PTC patients who require more extensive surgery, as well as careful follow-up and therapeutic strategy [5].

In this study, we evaluated the expression of the *BIRC5* gene encoding for the full length of survivin protein and its splice variants: survivin 2B and survivin delta Ex3 in thyroid cancer. The amount of these transcripts was analyzed in benign thyroid nodules, healthy thyroid tissues and carcinoma tissues.

Based on a quantitative RT-PCR technique we demonstrated a significantly higher expression of survivin and its splice variant – survivin delta Ex3 in malignant thyroid nodules as compared with benign tumors. Survivin 2B splice variant was expressed at a higher level in cancers but without statistical importance. Moreover, in our research survivin and both splice variants were expressed at significantly higher levels in the thyroid cancers than in the healthy thyroid tissues.

Our findings are in agreement with previous confirmatory reports of a consistently high expression of survivin in thyroid carcinoma tissues and its absence or downregulation in goiters. Survivin in that study was detected in 26% of goiters (4/15 by semi-quantitative RT-PCR, while its immunoreactivity observed in 87% of thyroid cancers was inversely correlated with the degree of their differentiation [7]. The previous and current research suggest, that survivin mRNA expression increases with any thyroid pathological process and it may vary due to size of thyroid sample cancer.

We also showed that the most prominent expression level of survivin was detected in undifferentiated thyroid cancers. Our findings are concordant with these previous publications [2], [5] They imply that survivin expression may be a marker of a worse prognosis of thyroid carcinoma.

The results of our study are also consistent with the observation regarding the expression of survivin and survivin delta Ex3 splice variant, which were shown to be significantly elevated in PTC when compared with benign tumors [5], [6]. The expression rate of survivin delta Ex3 has been especially shown to retain anti-apoptotic properties. In our study, we also observed a higher survivin delta Ex3 level of expression in thyroid malignant nodules. The relative amount of survivin delta Ex3 transcripts was much higher than the mRNA level of survivin, and survivin 2B.

In addition to an elevated expression level of survivin in many different types of cancer, the correlation between gene polymorphism and the risk of PTC was already documented. It has been shown that in the Iranian population, a polymorphism of the survivin gene at position-31 (G/C) is associated with the presence of more profound manifestations, including lymph node involvement, vascular involvement and multifocality [6]. It was also noted that the up-regulation of survivin protein expression may be enhanced in parallel with the transition towards a poorly differentiated phenotype in human thyroid carcinomas [6], [30].

In the thyroid cancer samples examined in our study, higher survivin 2B expression was found in tumor tissues at stages pT3/pT4 (*P = *0,0052). Moreover, a trend of survivin delta Ex3 overexpression in tumors staged pT3/pT4 compared to pT1 was observed (*P = *0,0548). Chen et al. previously showed a correlation between pT stages and the presence of lymph node metastases and distant metastases in thyroid carcinoma cases, while they did not observe any significant correlation in relation to gender, age and pathological subtype [7]. In our research, we also did not find any significant correlation in relation to age and gender. The comparison of survivin level and pathological subtypes was not evaluated because of small groups of different types of cancer.

The results of the present study document that the overexpression of survivin, and especially the survivin delta Ex3 splice variant, is a characteristic feature of thyroid cancers. Consequently, according to previous and current reports, the expression of survivin and its splice variants might help decide which patients with thyroid cancers require more aggressive treatment. Further studies on a larger population and a comparison of survivin expression with reference to tumor subtypes might help in our understanding of the role of survivin and its splice variant in thyroid cancers.
